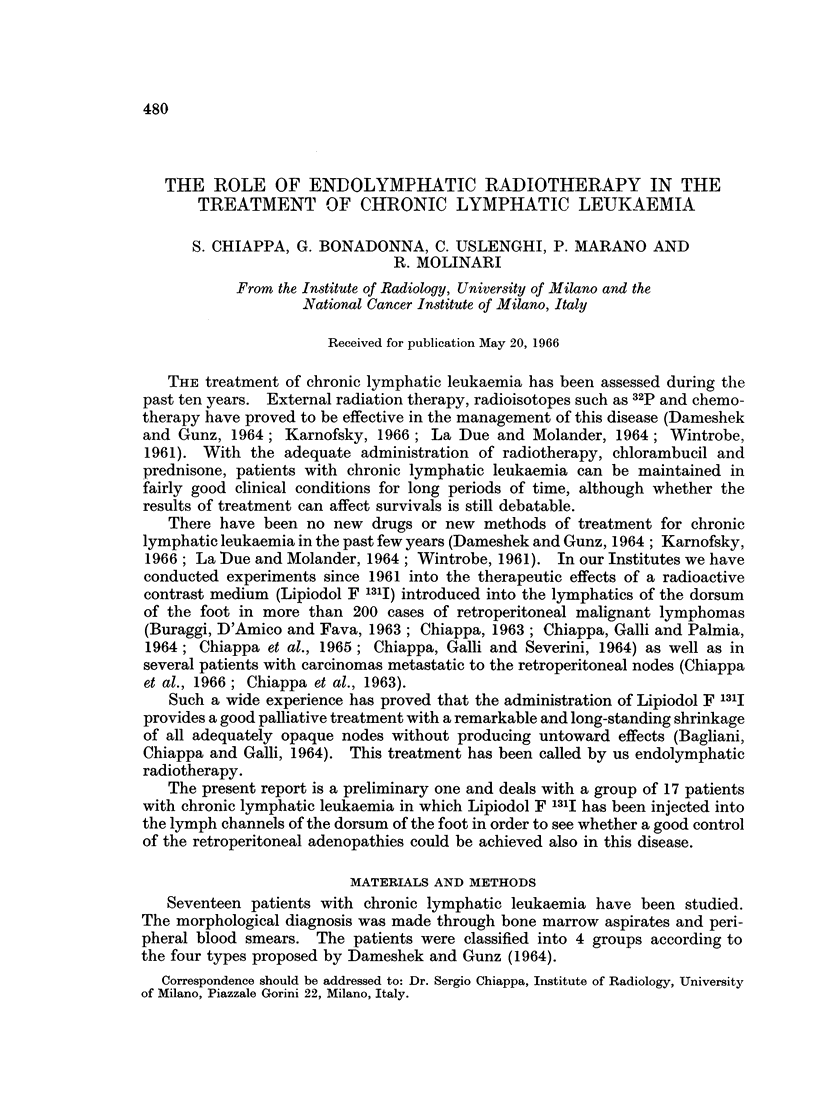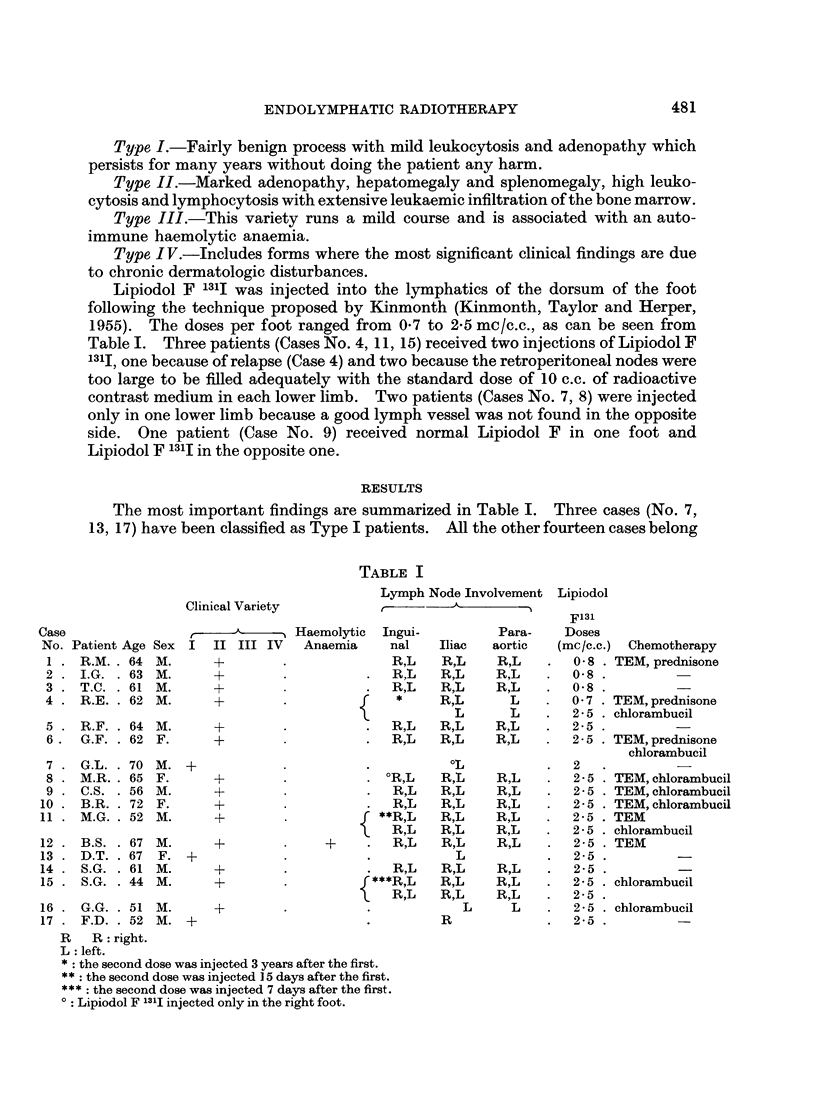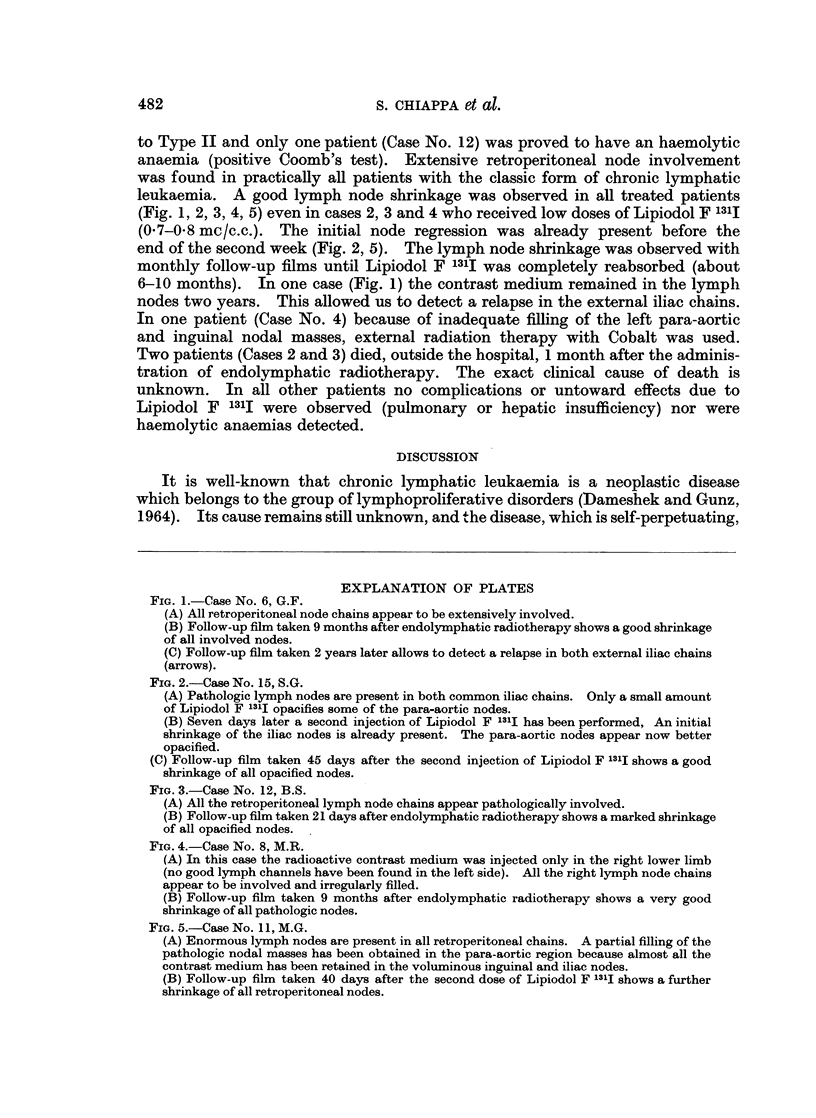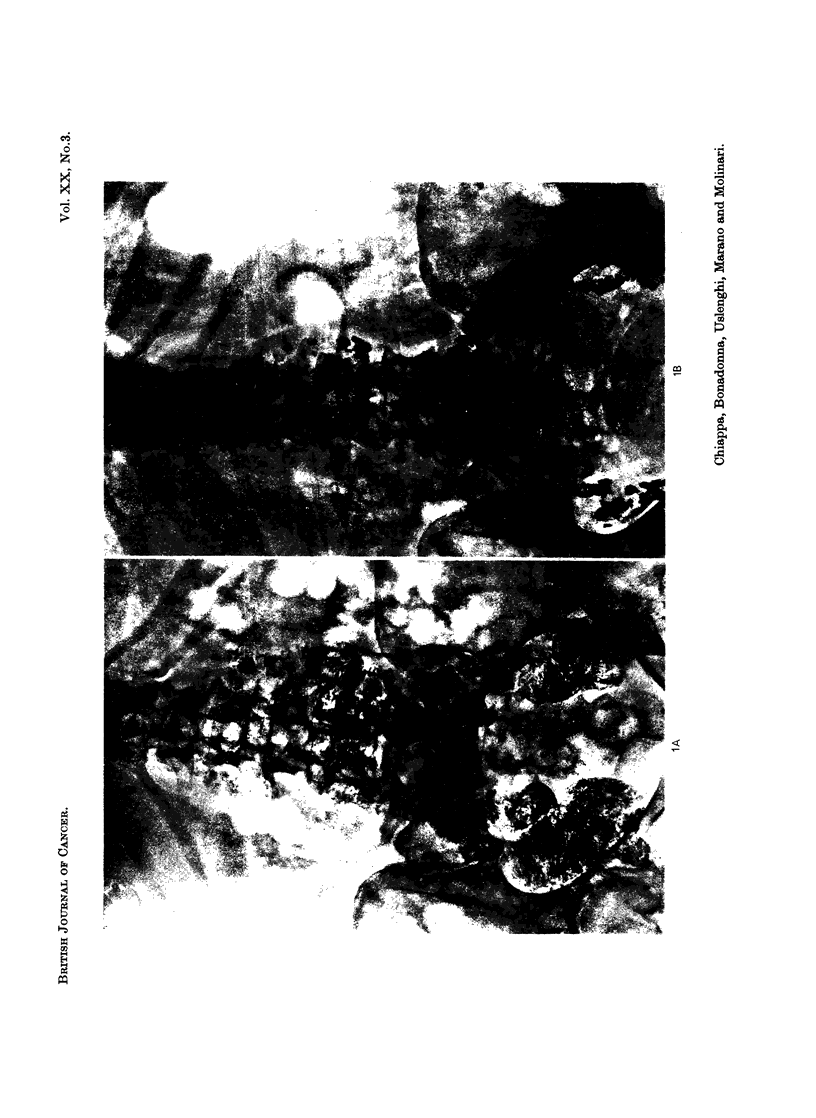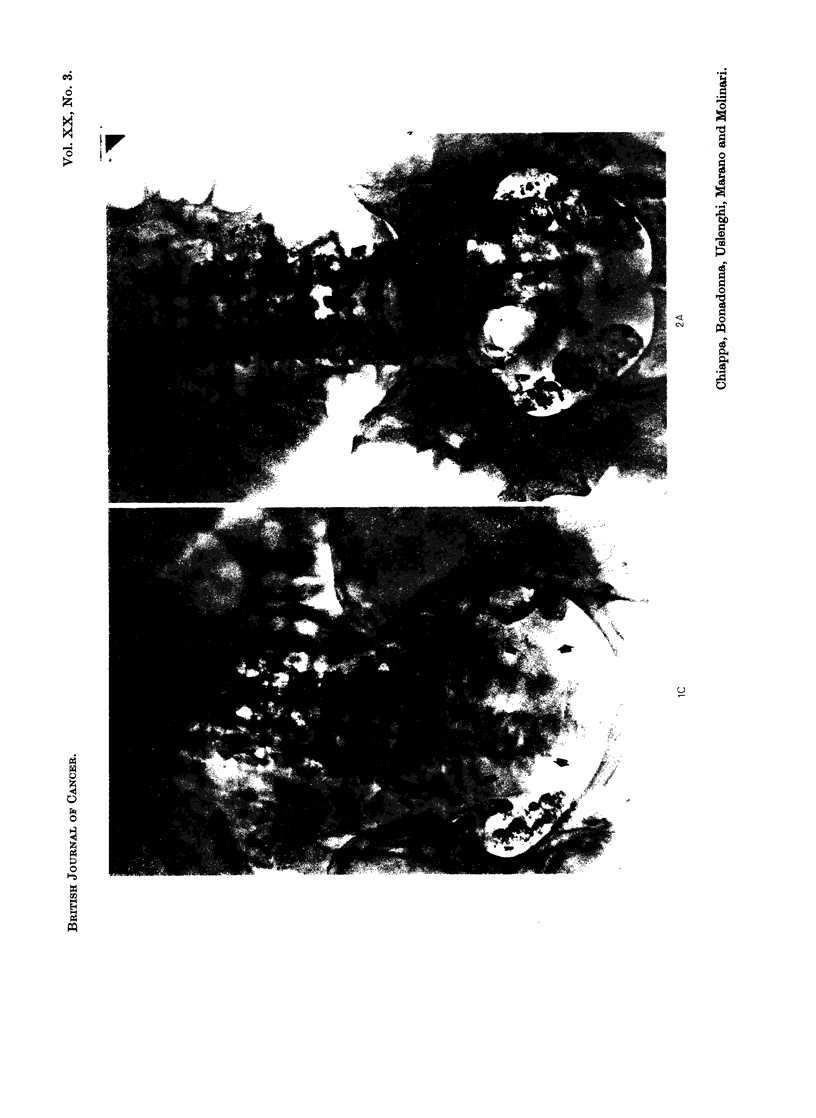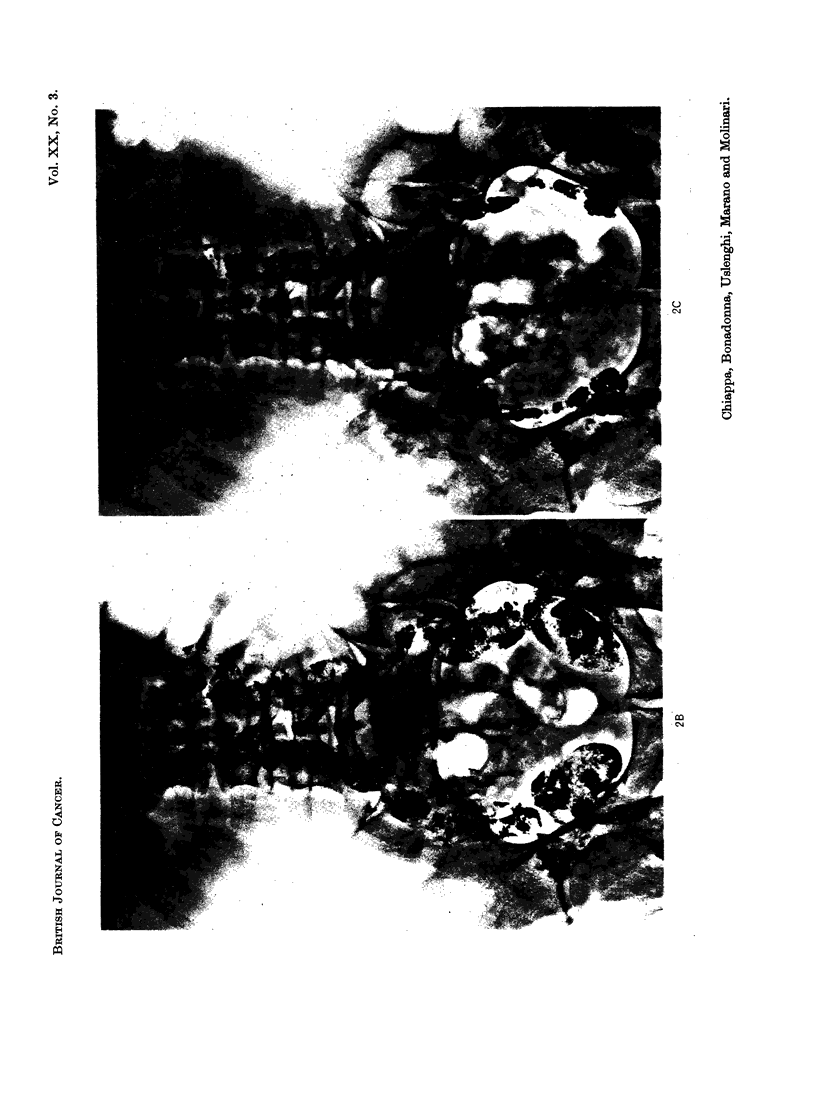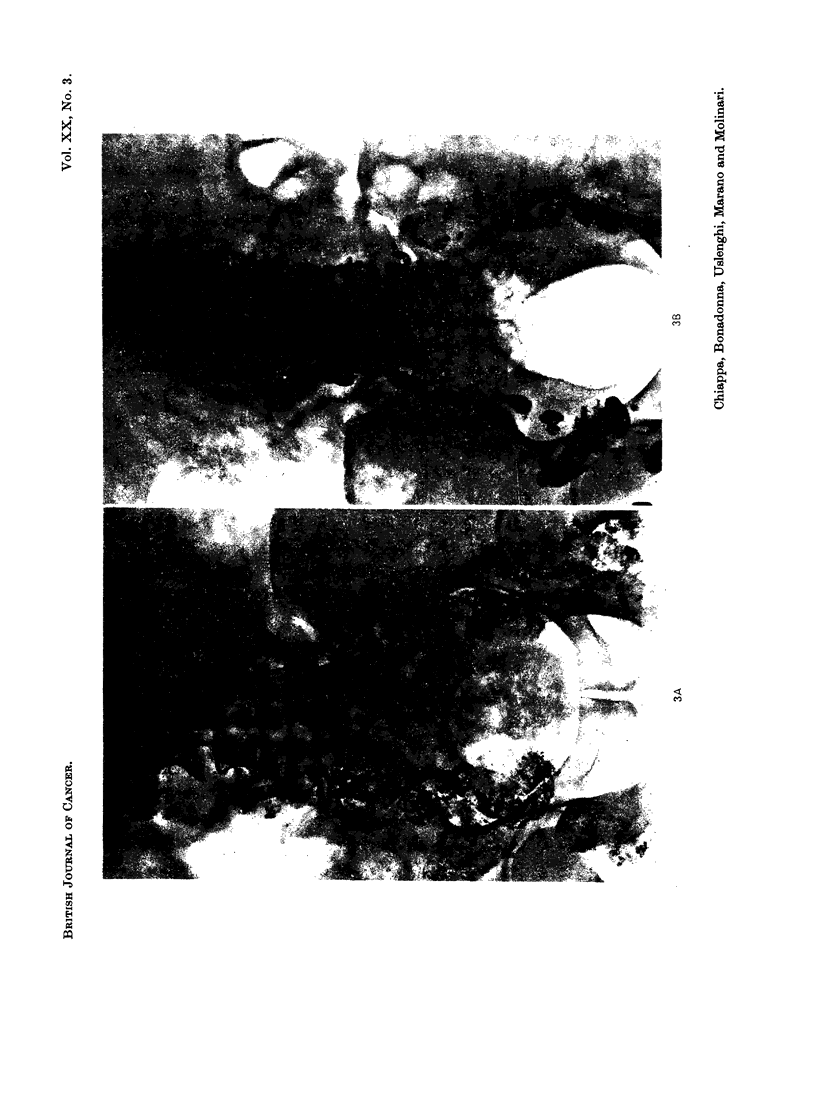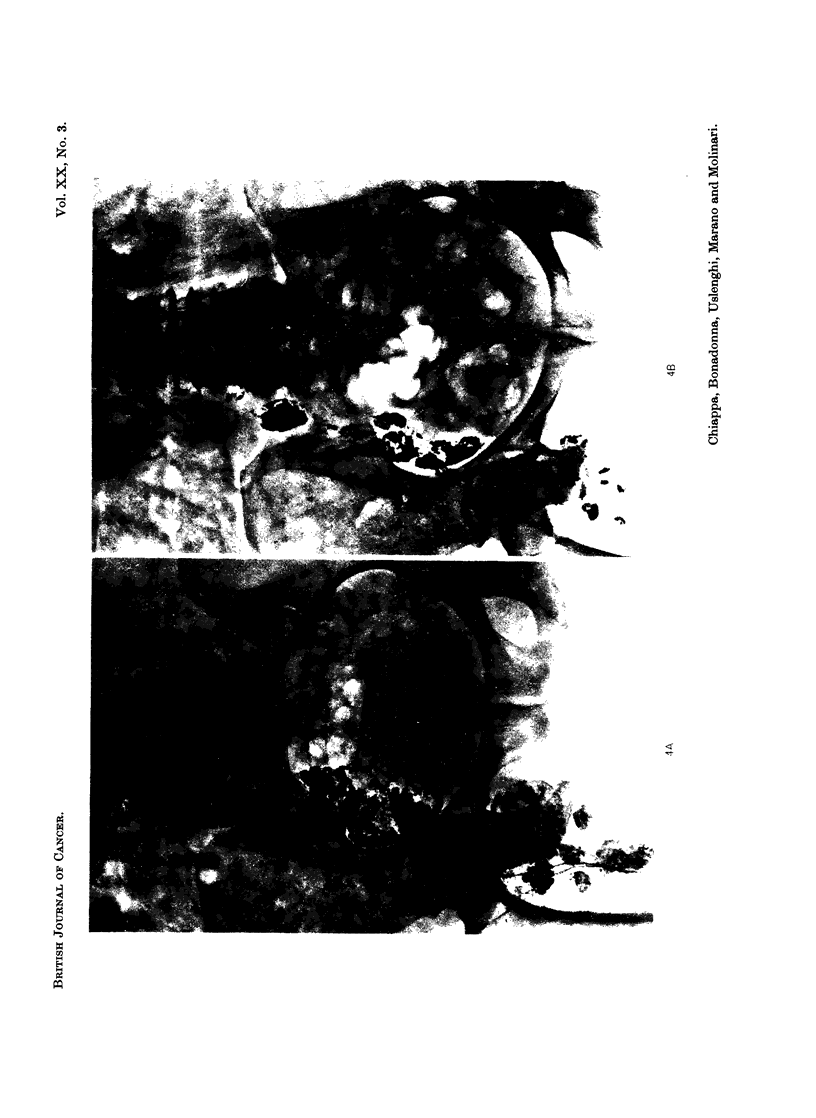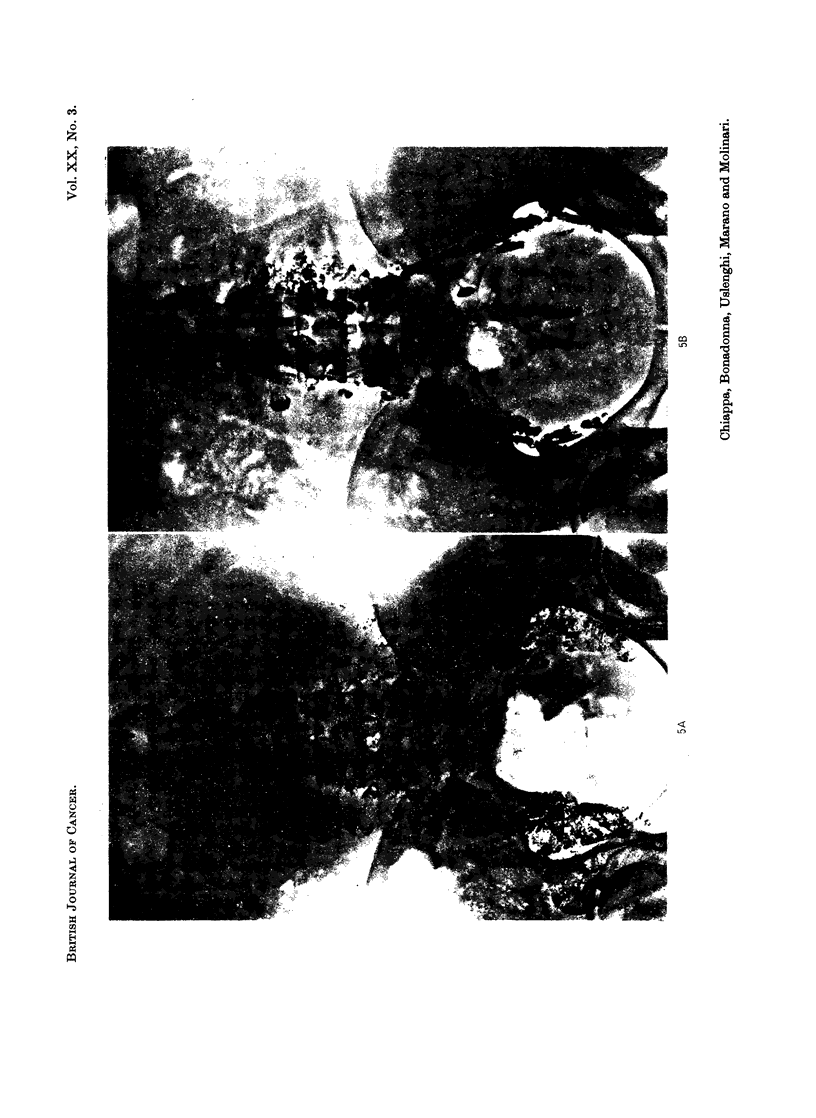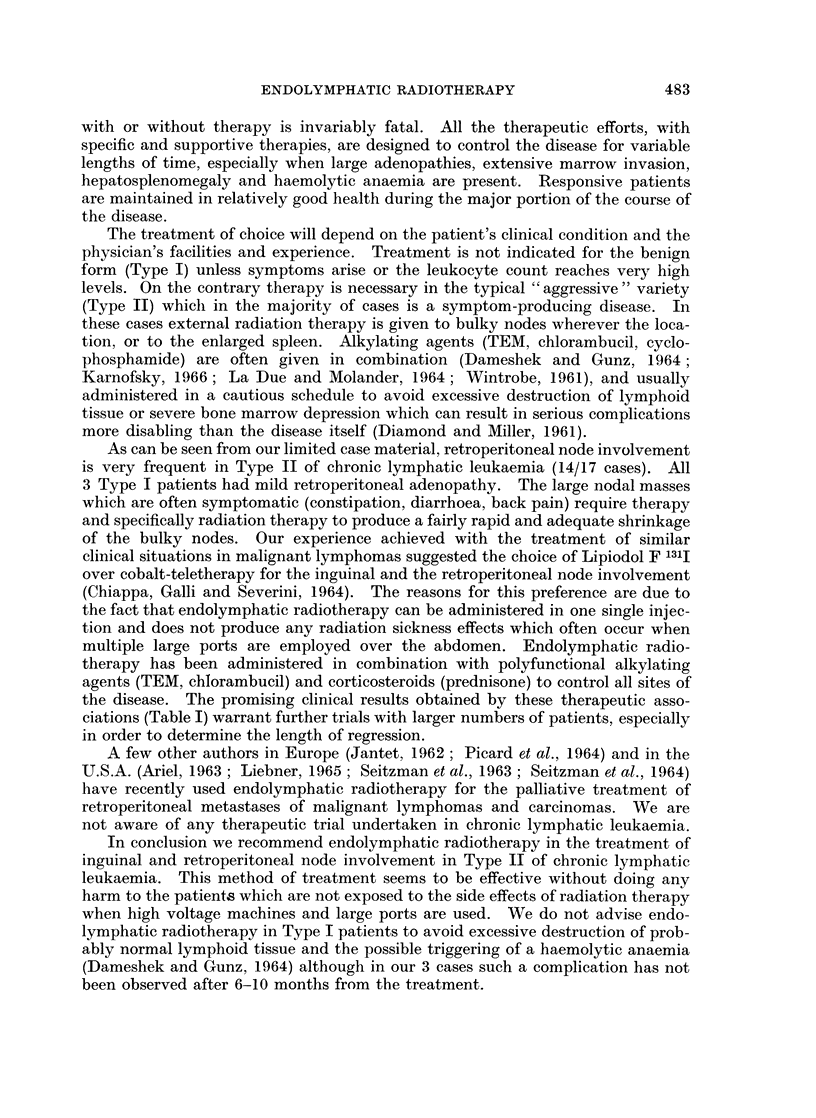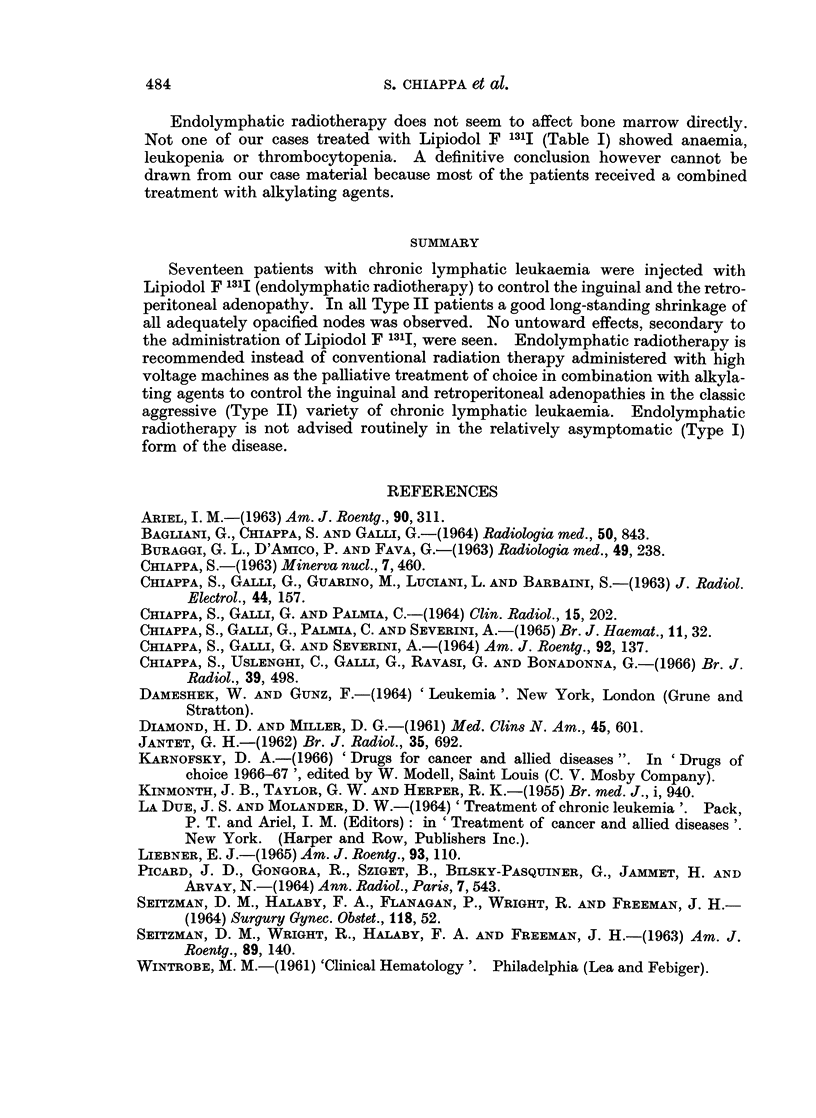# The role of endolymphatic radiotherapy in the treatment of chronic lymphatic leukaemia.

**DOI:** 10.1038/bjc.1966.59

**Published:** 1966-09

**Authors:** S. Chiappa, G. Bonadonna, C. Uslenghi, P. Marano, R. Molinari

## Abstract

**Images:**


### THE ROLE OF ENDOLYMPHATIC RADIOTHERAPY IN THE TREATMENT OF CHRONIC LYMPHATIC LEUKAEMIA

S. CHIAPPA, G. BONADONNA, C. USLENGHI, P. MARANO AND R. MOLINARI

*From the Institute of Radiology, University of Milano and the National Cancer Institute of Milano, Italy*

Received for publication May 20, 1966

THE treatment of chronic lymphatic leukaemia has been assessed during the past ten years. External radiation therapy, radioisotopes such as $^{32}P$ and chemotherapy have proved to be effective in the management of this disease (Dameshek and Gunz, 1964; Karnofsky, 1966; La Due and Molander, 1964; Wintrobe, 1961). With the adequate administration of radiotherapy, chlorambucil and prednisone, patients with chronic lymphatic leukaemia can be maintained in fairly good clinical conditions for long periods of time, although whether the results of treatment can affect survivals is still debatable.

There have been no new drugs or new methods of treatment for chronic lymphatic leukaemia in the past few years (Dameshek and Gunz, 1964; Karnofsky, 1966; La Due and Molander, 1964; Wintrobe, 1961). In our Institutes we have conducted experiments since 1961 into the therapeutic effects of a radioactive contrast medium (Lipiodol F $^{131}I$) introduced into the lymphatics of the dorsum of the foot in more than 200 cases of retroperitoneal malignant lymphomas (Buraggi, D'Amico and Fava, 1963; Chiappa, 1963; Chiappa, Galli and Palmia, 1964; Chiappa *et al.*, 1965; Chiappa, Galli and Severini, 1964) as well as in several patients with carcinomas metastatic to the retroperitoneal nodes (Chiappa *et al.*, 1966; Chiappa *et al.*, 1963).

Such a wide experience has proved that the administration of Lipiodol F $^{131}I$ provides a good palliative treatment with a remarkable and long-standing shrinkage of all adequately opaque nodes without producing untoward effects (Bagliani, Chiappa and Galli, 1964). This treatment has been called by us endolymphatic radiotherapy.

The present report is a preliminary one and deals with a group of 17 patients with chronic lymphatic leukaemia in which Lipiodol F $^{131}I$ has been injected into the lymph channels of the dorsum of the foot in order to see whether a good control of the retroperitoneal adenopathies could be achieved also in this disease.

### MATERIALS AND METHODS

Seventeen patients with chronic lymphatic leukaemia have been studied. The morphological diagnosis was made through bone marrow aspirates and peripheral blood smears. The patients were classified into 4 groups according to the four types proposed by Dameshek and Gunz (1964).

Correspondence should be addressed to: Dr. Sergio Chiappa, Institute of Radiology, University of Milano, Piazzale Gorini 22, Milano, Italy.

*Type I.*—Fairly benign process with mild leukocytosis and adenopathy which persists for many years without doing the patient any harm.

*Type II.*—Marked adenopathy, hepatomegaly and splenomegaly, high leukocytosis and lymphocytosis with extensive leukaemic infiltration of the bone marrow.

*Type III.*—This variety runs a mild course and is associated with an auto-immune haemolytic anaemia.

*Type IV.*—Includes forms where the most significant clinical findings are due to chronic dermatologic disturbances.

Lipiodol F $^{131}I$ was injected into the lymphatics of the dorsum of the foot following the technique proposed by Kinmonth (Kinmonth, Taylor and Herper, 1955). The doses per foot ranged from 0·7 to 2·5 mc/c.c., as can be seen from Table I. Three patients (Cases No. 4, 11, 15) received two injections of Lipiodol F $^{131}I$, one because of relapse (Case 4) and two because the retroperitoneal nodes were too large to be filled adequately with the standard dose of 10 c.c. of radioactive contrast medium in each lower limb. Two patients (Cases No. 7, 8) were injected only in one lower limb because a good lymph vessel was not found in the opposite side. One patient (Case No. 9) received normal Lipiodol F in one foot and Lipiodol F $^{131}I$ in the opposite one.

### RESULTS

The most important findings are summarized in Table I. Three cases (No. 7, 13, 17) have been classified as Type I patients. All the other fourteen cases belong

TABLE I

| Case No. | Patient | Age | Sex | Clinical Variety I | II | III | IV | Haemolytic Anaemia | Lymph Node Involvement: Inguinal | Iliac | Para-aortic | Lipiodol $F^{131}$ Doses (mc/c.c.) | Chemotherapy |
|---|---|---|---|---|---|---|---|---|---|---|---|---|---|
| 1 | R.M. | 64 | M. | | + | | | | R,L | R,L | R,L | 0·8 | TEM, prednisone |
| 2 | I.G. | 63 | M. | | + | | | | R,L | R,L | R,L | 0·8 | — |
| 3 | T.C. | 61 | M. | | + | | | | R,L | R,L | R,L | 0·8 | — |
| 4 | R.E. | 62 | M. | | + | | | | * | R,L | L | 0·7 | TEM, prednisone |
| | | | | | | | | | | L | L | 2·5 | chlorambucil |
| 5 | R.F. | 64 | M. | | + | | | | R,L | R,L | R,L | 2·5 | — |
| 6 | G.F. | 62 | F. | | + | | | | R,L | R,L | R,L | 2·5 | TEM, prednisone chlorambucil |
| 7 | G.L. | 70 | M. | + | | | | | | °L | | 2 | — |
| 8 | M.R. | 65 | F. | | + | | | | °R,L | R,L | R,L | 2·5 | TEM, chlorambucil |
| 9 | C.S. | 56 | M. | | + | | | | R,L | R,L | R,L | 2·5 | TEM, chlorambucil |
| 10 | B.R. | 72 | F. | | + | | | | R,L | R,L | R,L | 2·5 | TEM, chlorambucil |
| 11 | M.G. | 52 | M. | | + | | | | **R,L | R,L | R,L | 2·5 | TEM |
| | | | | | | | | | R,L | R,L | R,L | 2·5 | chlorambucil |
| 12 | B.S. | 67 | M. | | + | | | + | R,L | R,L | R,L | 2·5 | TEM |
| 13 | D.T. | 67 | F. | + | | | | | | L | | 2·5 | — |
| 14 | S.G. | 61 | M. | | + | | | | R,L | R,L | R,L | 2·5 | — |
| 15 | S.G. | 44 | M. | | + | | | | ***R,L | R,L | R,L | 2·5 | chlorambucil |
| | | | | | | | | | R,L | R,L | R,L | 2·5 | |
| 16 | G.G. | 51 | M. | | + | | | | | L | L | 2·5 | chlorambucil |
| 17 | F.D. | 52 | M. | + | | | | | | R | | 2·5 | — |

R R : right.
L : left.
* : the second dose was injected 3 years after the first.
** : the second dose was injected 15 days after the first.
*** : the second dose was injected 7 days after the first.
° : Lipiodol F $^{131}I$ injected only in the right foot.

to Type II and only one patient (Case No. 12) was proved to have an haemolytic anaemia (positive Coomb's test). Extensive retroperitoneal node involvement was found in practically all patients with the classic form of chronic lymphatic leukaemia. A good lymph node shrinkage was observed in all treated patients (Fig. 1, 2, 3, 4, 5) even in cases 2, 3 and 4 who received low doses of Lipiodol F $^{131}$I (0·7–0·8 mc/c.c.). The initial node regression was already present before the end of the second week (Fig. 2, 5). The lymph node shrinkage was observed with monthly follow-up films until Lipiodol F $^{131}$I was completely reabsorbed (about 6–10 months). In one case (Fig. 1) the contrast medium remained in the lymph nodes two years. This allowed us to detect a relapse in the external iliac chains. In one patient (Case No. 4) because of inadequate filling of the left para-aortic and inguinal nodal masses, external radiation therapy with Cobalt was used. Two patients (Cases 2 and 3) died, outside the hospital, 1 month after the administration of endolymphatic radiotherapy. The exact clinical cause of death is unknown. In all other patients no complications or untoward effects due to Lipiodol F $^{131}$I were observed (pulmonary or hepatic insufficiency) nor were haemolytic anaemias detected.

### DISCUSSION

It is well-known that chronic lymphatic leukaemia is a neoplastic disease which belongs to the group of lymphoproliferative disorders (Dameshek and Gunz, 1964). Its cause remains still unknown, and the disease, which is self-perpetuating,

---

### EXPLANATION OF PLATES

FIG. 1.—Case No. 6, G.F.

(A) All retroperitoneal node chains appear to be extensively involved.

(B) Follow-up film taken 9 months after endolymphatic radiotherapy shows a good shrinkage of all involved nodes.

(C) Follow-up film taken 2 years later allows to detect a relapse in both external iliac chains (arrows).

FIG. 2.—Case No. 15, S.G.

(A) Pathologic lymph nodes are present in both common iliac chains. Only a small amount of Lipiodol F $^{131}$I opacifies some of the para-aortic nodes.

(B) Seven days later a second injection of Lipiodol F $^{131}$I has been performed, An initial shrinkage of the iliac nodes is already present. The para-aortic nodes appear now better opacified.

(C) Follow-up film taken 45 days after the second injection of Lipiodol F $^{131}$I shows a good shrinkage of all opacified nodes.

FIG. 3.—Case No. 12, B.S.

(A) All the retroperitoneal lymph node chains appear pathologically involved.

(B) Follow-up film taken 21 days after endolymphatic radiotherapy shows a marked shrinkage of all opacified nodes.

FIG. 4.—Case No. 8, M.R.

(A) In this case the radioactive contrast medium was injected only in the right lower limb (no good lymph channels have been found in the left side). All the right lymph node chains appear to be involved and irregularly filled.

(B) Follow-up film taken 9 months after endolymphatic radiotherapy shows a very good shrinkage of all pathologic nodes.

FIG. 5.—Case No. 11, M.G.

(A) Enormous lymph nodes are present in all retroperitoneal chains. A partial filling of the pathologic nodal masses has been obtained in the para-aortic region because almost all the contrast medium has been retained in the voluminous inguinal and iliac nodes.

(B) Follow-up film taken 40 days after the second dose of Lipiodol F $^{131}$I shows a further shrinkage of all retroperitoneal nodes.

Chiappa, Bonadonna, Uslenghi, Marano and Molinari.

1C

2A

Chiappa, Bonadonna, Uslenghi, Marano and Molinari.

2B

2C

Chiappa, Bonadonna, Uslenghi, Marano and Molinari.

3A 3B

Chiappa, Bonadonna, Uslenghi, Marano and Molinari.

4A 4B

Chiappa, Bonadonna, Uslenghi, Marano and Molinari.

5A 5B

Chiappa, Bonadonna, Uslenghi, Marano and Molinari.

with or without therapy is invariably fatal. All the therapeutic efforts, with specific and supportive therapies, are designed to control the disease for variable lengths of time, especially when large adenopathies, extensive marrow invasion, hepatosplenomegaly and haemolytic anaemia are present. Responsive patients are maintained in relatively good health during the major portion of the course of the disease.

The treatment of choice will depend on the patient's clinical condition and the physician's facilities and experience. Treatment is not indicated for the benign form (Type I) unless symptoms arise or the leukocyte count reaches very high levels. On the contrary therapy is necessary in the typical "aggressive" variety (Type II) which in the majority of cases is a symptom-producing disease. In these cases external radiation therapy is given to bulky nodes wherever the location, or to the enlarged spleen. Alkylating agents (TEM, chlorambucil, cyclophosphamide) are often given in combination (Dameshek and Gunz, 1964; Karnofsky, 1966; La Due and Molander, 1964; Wintrobe, 1961), and usually administered in a cautious schedule to avoid excessive destruction of lymphoid tissue or severe bone marrow depression which can result in serious complications more disabling than the disease itself (Diamond and Miller, 1961).

As can be seen from our limited case material, retroperitoneal node involvement is very frequent in Type II of chronic lymphatic leukaemia (14/17 cases). All 3 Type I patients had mild retroperitoneal adenopathy. The large nodal masses which are often symptomatic (constipation, diarrhoea, back pain) require therapy and specifically radiation therapy to produce a fairly rapid and adequate shrinkage of the bulky nodes. Our experience achieved with the treatment of similar clinical situations in malignant lymphomas suggested the choice of Lipiodol F $^{131}$I over cobalt-teletherapy for the inguinal and the retroperitoneal node involvement (Chiappa, Galli and Severini, 1964). The reasons for this preference are due to the fact that endolymphatic radiotherapy can be administered in one single injection and does not produce any radiation sickness effects which often occur when multiple large ports are employed over the abdomen. Endolymphatic radiotherapy has been administered in combination with polyfunctional alkylating agents (TEM, chlorambucil) and corticosteroids (prednisone) to control all sites of the disease. The promising clinical results obtained by these therapeutic associations (Table I) warrant further trials with larger numbers of patients, especially in order to determine the length of regression.

A few other authors in Europe (Jantet, 1962; Picard *et al.*, 1964) and in the U.S.A. (Ariel, 1963; Liebner, 1965; Seitzman *et al.*, 1963; Seitzman *et al.*, 1964) have recently used endolymphatic radiotherapy for the palliative treatment of retroperitoneal metastases of malignant lymphomas and carcinomas. We are not aware of any therapeutic trial undertaken in chronic lymphatic leukaemia.

In conclusion we recommend endolymphatic radiotherapy in the treatment of inguinal and retroperitoneal node involvement in Type II of chronic lymphatic leukaemia. This method of treatment seems to be effective without doing any harm to the patients which are not exposed to the side effects of radiation therapy when high voltage machines and large ports are used. We do not advise endolymphatic radiotherapy in Type I patients to avoid excessive destruction of probably normal lymphoid tissue and the possible triggering of a haemolytic anaemia (Dameshek and Gunz, 1964) although in our 3 cases such a complication has not been observed after 6–10 months from the treatment.

Endolymphatic radiotherapy does not seem to affect bone marrow directly. Not one of our cases treated with Lipiodol F $^{131}$I (Table I) showed anaemia, leukopenia or thrombocytopenia. A definitive conclusion however cannot be drawn from our case material because most of the patients received a combined treatment with alkylating agents.

### SUMMARY

Seventeen patients with chronic lymphatic leukaemia were injected with Lipiodol F $^{131}$I (endolymphatic radiotherapy) to control the inguinal and the retroperitoneal adenopathy. In all Type II patients a good long-standing shrinkage of all adequately opacified nodes was observed. No untoward effects, secondary to the administration of Lipiodol F $^{131}$I, were seen. Endolymphatic radiotherapy is recommended instead of conventional radiation therapy administered with high voltage machines as the palliative treatment of choice in combination with alkylating agents to control the inguinal and retroperitoneal adenopathies in the classic aggressive (Type II) variety of chronic lymphatic leukaemia. Endolymphatic radiotherapy is not advised routinely in the relatively asymptomatic (Type I) form of the disease.

### REFERENCES

ARIEL, I. M.—(1963) *Am. J. Roentg.*, **90**, 311.
BAGLIANI, G., CHIAPPA, S. AND GALLI, G.—(1964) *Radiologia med.*, **50**, 843.
BURAGGI, G. L., D'AMICO, P. AND FAVA, G.—(1963) *Radiologia med.*, **49**, 238.
CHIAPPA, S.—(1963) *Minerva nucl.*, **7**, 460.
CHIAPPA, S., GALLI, G., GUARINO, M., LUCIANI, L. AND BARBAINI, S.—(1963) *J. Radiol. Electrol.*, **44**, 157.
CHIAPPA, S., GALLI, G. AND PALMIA, C.—(1964) *Clin. Radiol.*, **15**, 202.
CHIAPPA, S., GALLI, G., PALMIA, C. AND SEVERINI, A.—(1965) *Br. J. Haemat.*, **11**, 32.
CHIAPPA, S., GALLI, G. AND SEVERINI, A.—(1964) *Am. J. Roentg.*, **92**, 137.
CHIAPPA, S., USLENGHI, C., GALLI, G., RAVASI, G. AND BONADONNA, G.—(1966) *Br. J. Radiol.*, **39**, 498.
DAMESHEK, W. AND GUNZ, F.—(1964) ' Leukemia '. New York, London (Grune and Stratton).
DIAMOND, H. D. AND MILLER, D. G.—(1961) *Med. Clins N. Am.*, **45**, 601.
JANTET, G. H.—(1962) *Br. J. Radiol.*, **35**, 692.
KARNOFSKY, D. A.—(1966) ' Drugs for cancer and allied diseases ". In ' Drugs of choice 1966–67 ', edited by W. Modell, Saint Louis (C. V. Mosby Company).
KINMONTH, J. B., TAYLOR, G. W. AND HERPER, R. K.—(1955) *Br. med. J.*, i, 940.
LA DUE, J. S. AND MOLANDER, D. W.—(1964) ' Treatment of chronic leukemia '. Pack, P. T. and Ariel, I. M. (Editors) : in ' Treatment of cancer and allied diseases '. New York. (Harper and Row, Publishers Inc.).
LIEBNER, E. J.—(1965) *Am. J. Roentg.*, **93**, 110.
PICARD, J. D., GONGORA, R., SZIGET, B., BILSKY-PASQUINER, G., JAMMET, H. AND ARVAY, N.—(1964) *Ann. Radiol., Paris*, **7**, 543.
SEITZMAN, D. M., HALABY, F. A., FLANAGAN, P., WRIGHT, R. AND FREEMAN, J. H.—(1964) *Surgury Gynec. Obstet.*, **118**, 52.
SEITZMAN, D. M., WRIGHT, R., HALABY, F. A. AND FREEMAN, J. H.—(1963) *Am. J. Roentg.*, **89**, 140.
WINTROBE, M. M.—(1961) 'Clinical Hematology '. Philadelphia (Lea and Febiger).